# Case Report: Post-splenectomy bulky pelvic splenosis in an adolescent with hereditary spherocytosis

**DOI:** 10.3389/fped.2025.1581533

**Published:** 2025-04-28

**Authors:** Benedetta Elena Di Majo, Nicolò Peccatori, Alessandra Inzoli, Luca Degrate, Marta Jaconi, Michele Ratti, Alessandra Casiraghi, Giulia Maria Ferrari, Debora Sala, Andrea Biondi, Paolo Passoni, Paola Corti

**Affiliations:** ^1^ Department of Pediatrics, Fondazione IRCCS San Gerardo dei Tintori, Monza, Italy; ^2^ School of Medicine and Surgery, University of Milano-Bicocca, Milan, Italy; ^3^ Tettamanti Center, Fondazione IRCCS San Gerardo dei Tintori, Monza, Italy; ^4^ Department of Gynecology and Obstetrics, Fondazione IRCCS San Gerardo dei Tintori, Monza, Italy; ^5^ Department of General and Emergency Surgery, Fondazione IRCCS San Gerardo dei Tintori, Monza, Italy; ^6^ Department of Pathology, Fondazione IRCCS San Gerardo dei Tintori, Monza, Italy; ^7^ Department of Radiology, Fondazione IRCCS San Gerardo dei Tintori, Monza, Italy

**Keywords:** splenosis, splenectomy, hereditary spherocytosis, spleen, pediatric

## Abstract

Splenectomy is a well-established therapeutic approach for pediatric hematologic disorders, especially in the case of hereditary spherocytosis (HS). In addition to the commonly acknowledged short- and long-term infectious and thrombotic complications, also splenosis represents a rare but noteworthy complication of splenectomy. Splenosis is characterized by the auto-transplantation and growth of splenic tissue in ectopic locations, following trauma or splenectomy. This condition can mimic malignancies, posing diagnostic challenges. We report the case of a 16-year-old girl with HS who presented with fever, abdominal pain, and a history of laparoscopic splenectomy ten years early. Imaging revealed a vascularized pelvic mass, initially suspected to be malignant. Diagnostic laparoscopy and histopathological analysis confirmed the mass as pelvic splenosis. The patient was asymptomatic, prompting a conservative management approach with regular follow-up. This case highlights the importance of considering splenosis in differential diagnoses for pelvic masses in patients with prior splenectomy, to ensure appropriate management and avoid unnecessary interventions.

## Introduction

Splenectomy represents a potential therapeutic strategy for the management of various pediatric hematologic disorders. Particularly in hereditary spherocytosis (HS), the effectiveness of splenectomy has been well documented, resulting in the correction of anemia and hemolytic markers in most of the patients (1, 2). International guidelines for the management of HS with specific recommendations for splenectomy indication are available, tailored to the severity of the disease (2, 3). Nevertheless, splenectomy is associated with the risk of both short- and long-term infectious and thrombotic complications, with the overwhelming post-splenectomy infection (OPSI) being the most recognized and feared complication (4). Although rare, splenosis also represents a possible complication occurring also several years after splenectomy. Splenosis is, thus, defined as an auto-transplantation of splenic tissue to an ectopic location, which can occur after splenic trauma or splenectomy. Its diagnosis is often challenging, as it may be an incidental finding or presents with a wide spectrum of different clinical manifestations mimicking various other conditions, including malignancies (5–8). Herein, we report a challenging case of extensive post-splenectomy pelvic splenosis in an adolescent with HS.

## Case description

**Figure 1:**
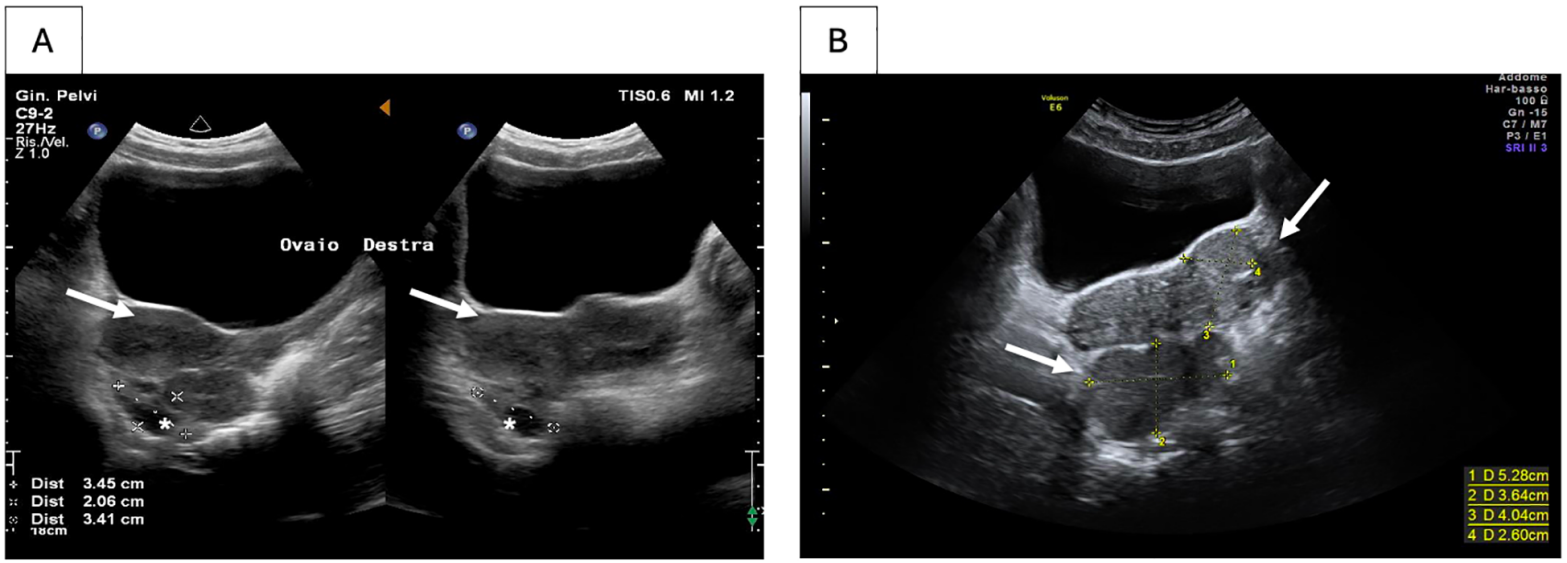
Ultrasound of the pelvic splenosis. Splenosis **(A)** at diagnosis and **(B)** six months after diagnosis. White arrows indicate the ectopic splenic tissue. Right ovary is shown by the *.

**Figure 2:**
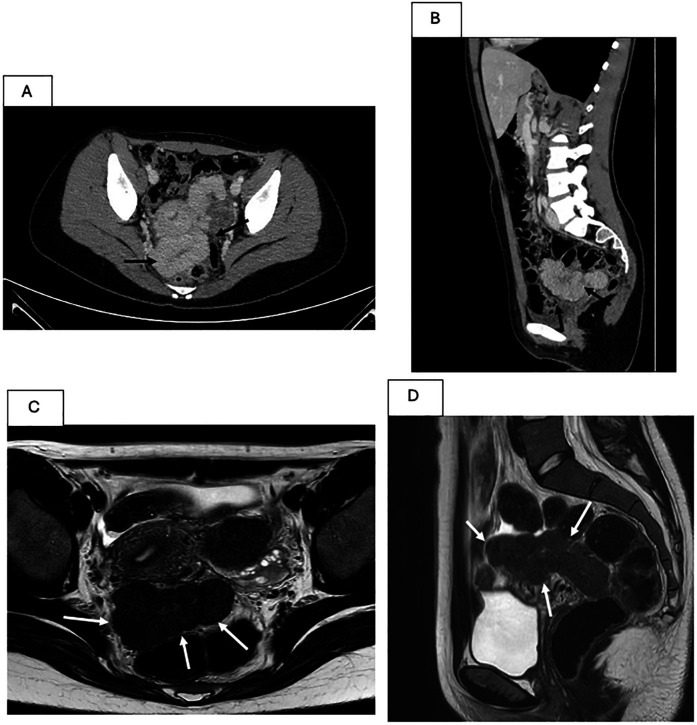
CT and MRI aspect of the pelvic splenosis. **(A,B)** CT scan. A serpiginous formation with dimensions of 120 × 60 × 55 mm, with homogeneous contrast enhancement in the portal-venous phase, is indicated by the black arrows in axial **(A)** and sagittal **(B)** plane. **(C,D)** MRI. Multilobulated formation with regular margins and confluent pseudonodular components with elongated morphology (axial dimensions of 10 × 4.7 cm and a craniocaudal extension of approximately 6.3 cm) is indicated by the white arrows, located between the uterus and the rectum in axial T2-weighted **(C)** and sagittal T2-weighted **(D)** images.

A 16-year-old girl with HS presented to the pediatric emergency department with fever (39.3°C), sore throat, and mild abdominal pain. She had a history of laparoscopic splenectomy and cholecystectomy at the age of 6 years, due to recurrent hemolytic crises requiring frequent red blood cell transfusions and a history of cholelithiasis in a form of severe hereditary spherocytosis. The hematologic outcome post-splenectomy was optimal, with no further need for transfusions or new episodes of hemolytic or aplastic crises since then. Her immunizations were up to date. On physical examination, hypertrophic pharyngitis and widespread tenderness in the lower abdominal quadrants without signs of guarding were observed. The remainder of her physical examination was unremarkable. Given the risk of severe infection in a febrile patient with asplenia, blood tests were performed, and empiric broad-spectrum antibiotic therapy with ceftriaxone was promptly started. The complete blood count showed a moderate neutrophil leukocytosis (white blood cell count -WBC- of 19.0 × 10^9^/L; neutrophils 15.2 × 10^9^/L) with normal hemoglobin level and platelets count (121 g/L and 415 × 10^9^/L, respectively). Serum chemistry was unremarkable except for moderate elevation of c-reactive protein -CRP- (90 mg/L; normal values <5); no relevant chronic hemolysis was present. An abdominal ultrasound was performed and, unexpectedly, revealed the presence of an extensive pelvic mass. The hypoechoic, highly vascularized (Color Score 3, CS3) lesion, measuring 7.3 × 2.2 × 5.7 cm, with indistinct margins was located anteriorly to the left ovary and seemingly separable from it. A clear cleavage plane from the uterus and the bladder was undetectable (Figure 1). The patient was admitted for further investigations. A Streptococcal pharyngitis was diagnosed by detecting group A *streptococcus pyogenes* (GAS) in the throat swab culture test, and oral antibiotic therapy switch was done with rapid resolution of the infection. The abdominal pain resolved spontaneously. To better characterize the pelvic mass, a contrast-enhanced computed tomography scan (CT) and a magnetic resonance imaging (MRI) of the abdomen and pelvis were performed. The CT scan confirmed the dimensions of the pelvic mass, apparently not attributable to the uterus or the adnexa; no retroperitoneal lymphadenopathy was detected (Figure 2), while the MRI provided a clearer visualization of the multilobulated mass revealing confluent pseudo-nodular components located in the hypogastric region between the uterus and the left ovary (Figure 2). The signal on T2-weighted (T2W) sequences was homogeneously hypointense, while on T1-weighted (T1W) sequences was intermediate with mild homogeneous enhancement after contrast administration during the arterial, portal-venous, and delayed phases. No adipose cleavage plane was observed between the mass and the left ovary or the uterine body fundus. The mass displaced the uterus to the right, and the right ovary posteriorly. The abdominal CT scan characteristics in the acquired portal-venous phase and the previous splenectomy suggested a possible diagnosis of pelvic splenosis; the arterial phase was not acquired to minimize radiation exposure. However, the MRI images did not appear typical for splenic parenchyma, and the T2W signal intensity and location of the mass were also compatible with the differential diagnosis of broad ligament leiomyoma, although with an atypical morphology. Serum tumor markers including, lactate dehydrogenase (LDH), alpha-fetoprotein (AFP), carcinoembryonic antigen (CEA), cancer antigen 125 (CA-125), and carbohydrate antigen 19-9 (CA-19.9) were normal.

**Figure 3:**
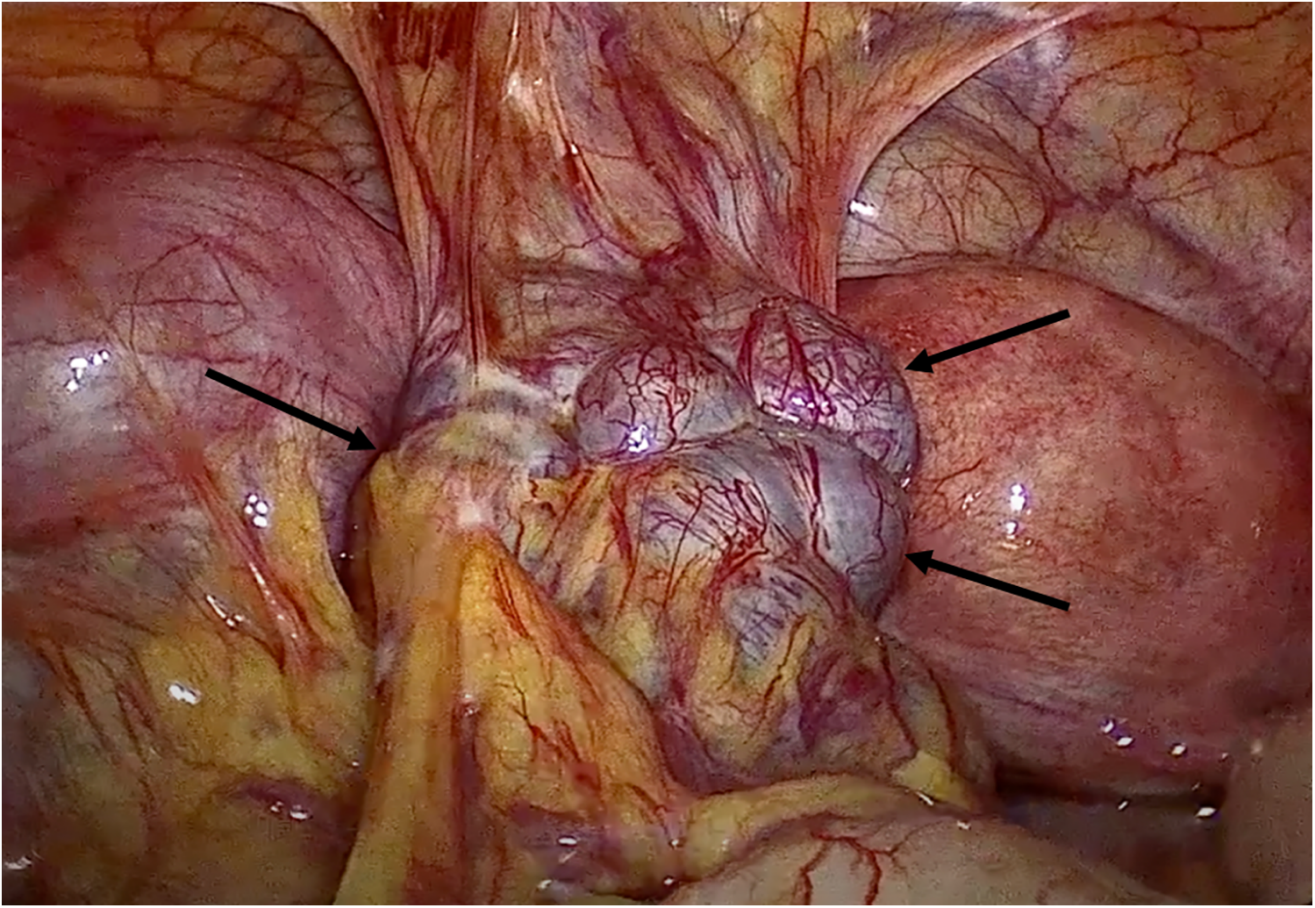
Intraoperative laparoscopic view of the pelvic splenosis. The multilobulated pelvic mass (black arrows) showed tense adhesions with pelvic wall, bladder, uterus and sigmoid colon.

Considering the different diagnostic hypotheses, a biopsy was planned. Due to the lesion's high vascularization and the associated hemorrhagic risk, a diagnostic laparoscopy was chosen. Intraoperatively, the multilobulated mass was in the pelvic region (Figure 3) anteriorly to the uterus, exerting significant retraction on the posterior wall of the bladder. Laterally, it was in contact with the left fallopian tube, which appeared edematous, thickened, and dilated. The left ovary was not visible due to the presence of the mass, which adhered to the lateral pelvic wall. The sigmoid colon formed dense adhesions with the mass, extending to the pouch of Douglas. Furthermore, two other sub-centimetric analogous masses on the sigmoid colon and several lesions on the anterior abdominal wall were noted.

**Figure 4:**
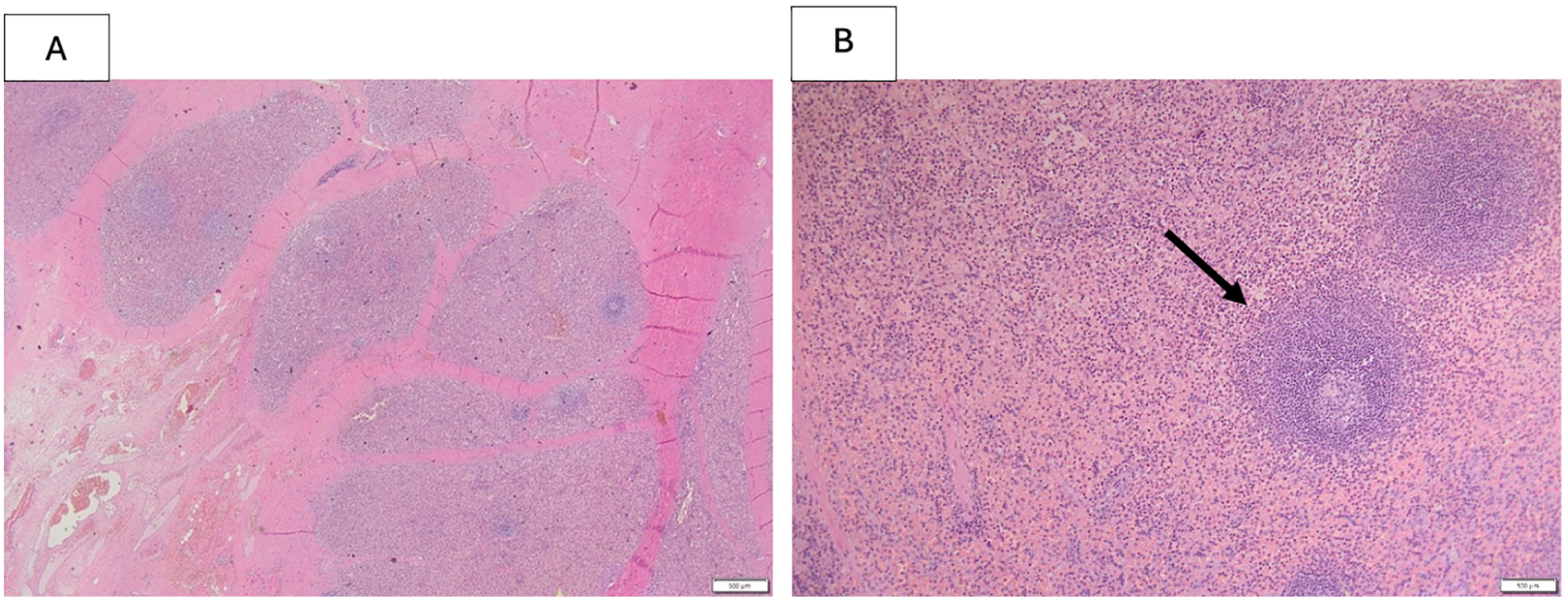
Histopathological features of splenic tissue biopsy including fibrous capsule, the black arrow shows a perivascular lymphocyte cuff. Hematoxylin and Eosin (H&E) staining, 500 um.

The histopathological trans-operative frozen section analysis of the biopsy sample revealed splenic tissue, confirming the diagnosis of pelvic splenosis (Figure 4). Due to the risk of intestinal resection related to the dense adhesions of the mass with the pelvic surrounding structures, and considering the patient's asymptomatic condition, it was decided to withhold surgical treatment. Post-operative course was uneventful. The echographic follow-up, set annually, showed a substantial stability of the lesion and currently the girl remains asymptomatic 1 year after the diagnosis. The patient and her parents have been informed about the risk of potential further growth of the mass. In the event of a pregnancy, she has been advised to notify the obstetrical team about her condition**.**

## Discussion

We present the case of an adolescent girl with HS diagnosed with a bulky pelvic splenosis 10 years after splenectomy. Splenectomy is indicated in severe transfusion-dependent HS, in moderate forms of HS with recurrent hemolytic crisis requiring frequent blood transfusions and in severe cytopenia secondary to hypersplenism (2). In our case, laparoscopic splenectomy was performed due to recurrent hemolytic and aplastic crises. Laparoscopic splenectomy is currently the standard operative technique for elective splenectomy in hereditary hemolytic anemias, due to several advantages including low surgical mortality, reduced complications, shorter time of hospitalization, better cosmetic outcome and lower cost as compared to open splenectomy (2). The spleen, laparoscopically removed, is placed into a surgical bag (endobag) to allow tissue fragmentation while preventing the spillage of splenic tissue in the abdominal cavity.

Splenosis is defined as an acquired and benign condition characterized by the auto-implantation and uncontrolled growth of splenic tissue through various anatomical compartments of the body. The most frequent sites of splenosis are the abdominal and pelvic cavities, but it has been occasionally described in other locations, including the liver and the thorax (5, 9, 10, 11). Splenosis typically arises following traumatic rupture of the spleen, where its incidence varies between 16% and 67% of the cases (13). In case of splenic rupture, splenosis can be found in up to 65% or 72% of cases depending on the study (11, 14). Most cases result from the local dissemination of splenic tissue into the abdomen compartment, and in rare specific cases, namely intrahepatic and thoracic splenosis, from the intravascular migration of splenic cells to ectopic locations (5, 8, 10). Apparently, no surgical complications, i.e., ruptures of the endobag, were observed during splenectomy in our patient. Nonetheless the way of dissemination of splenosis inside the abdominal cavity, from the left lower quadrant where the endobag was inserted, suggests that an intraoperatitve spillage of spleen cells happened through a microscopic perforation of the endobag.

Depending on its localization, splenosis can remain asymptomatic for an extended period, with its diagnosis often being incidental, as it was in our case. Splenic tissue growth rate is usually slow, with a median interval between the triggering trauma and the development of splenosis of approximately 10 years (9). Although very rare, its real incidence is not known, and it is possibly underestimated. It could be incidentally diagnosed during surgery or autopsy (14).

Ectopic splenic tissue characteristic of splenosis must be distinguished from accessory spleens, which are common, benign congenital deposits of functional splenic tissue and are estimated to occur in 10%–30% of the population (12).

A systematic review of the literature of pelvic splenosis was recently conducted by Peitsidis et al., which identified 85 reported cases and summarized their clinical features and treatment management (8). In this series, mean age of the patients was 51.7 years (17–74 years) and almost 80% of patients were of female gender. An emergency presentation was noted in approximately one third of patients, with abdominal pain as the most frequent symptom. In most of the cases, pelvic splenosis followed splenectomy for traffic injuries (56%) or abdominal trauma (32.9%). Only one case of splenosis was reported after elective splenectomy in HS. Interestingly, it was reported that women can manifest gynecological symptoms with dyspareunia, dysmenorrhea and infertility being the most frequently described. Thus, in female patients the differential diagnosis may become particularly challenging, misleading to endometriosis or adnexal neoplasms (15). As observed in our patient, splenosis can become invasive and may potentially contribute to infertility in female patients. Mollo et al. reported a case of pelvic inflammatory reaction due to ectopic splenosis implants in a female patient who had undergone splenectomy 20 years earlier for transfusion-dependent beta-thalassemia. Fimbrial biopsy was not performed, therefore a direct association between pelvic splenosis and infertility could not be determined. Nonetheless, it is known that surgery can lead to complications related to tenaciously adhered splenosis to adjacent organs, and surgical decision must be properly weighed (16).

On radiology findings, splenosis can mimic various pathologic entities, including primary malignancy or metastatic disease (12). On CT scan, splenosis exhibits similar features of normal splenic tissue. Similarly, MRI shows splenic nodules with intensity and enhancement resembling normal spleen, offering the advantages of no radiation, multiparametric approach and excellent soft tissue contrast for improved specificity compared to CT imaging. Nevertheless, the diagnostic management of abdominal splenosis using these conventional radiological techniques often reveals significant limitations and gives rise to diagnostic controversies, as occurred in our case.

Nuclear scintigraphy with Technetium 99 (Tc-99) is the gold standard non-invasive diagnostic method for splenosis in which Tc-99 sulfurous colloid is absorbed in the reticuloendothelial system and detects ectopic splenic tissues (8). When Tc-99 sulfur colloid fails to confirm the presence of splenic tissue, heat-damaged technetium-99-tagged red blood cells (RBC) is the preferred noninvasive diagnostic tool, offering greater sensitivity and specificity than sulfur colloid scintigraphy for confirming splenosis (9). The use of 99mTc-labeled colloid scintigraphy in conjunction with single-photon emission computed tomography (SPECT) enables precise three-dimensional localization. Moreover, 99mTc-labeled colloid SPECT/CT has been documented as a valuable diagnostic tool for identifying intrapancreatic accessory spleens. In this modality, SPECT provides functional or tissue-specific insights, while CT facilitates accurate anatomical localization, collectively enhancing diagnostic accuracy (17). Despite advanced radiological techniques, a biopsy is often necessary to certainly diagnose splenosis and rule out differential diagnoses.

While biopsy of the spleen is typically avoided due to bleeding risks, biopsy of splenic implants is considered safe when easily accessible. Importantly, the blood supply of ectopic splenic tissue in splenosis deeply differs from the proper spleen. Splenosis implants derive their blood supply from surrounding tissue vessels rather than the splenic artery. This vascular difference likely contributes to the lower bleeding risk associated with biopsies of ectopic splenic tissue, making the procedure a feasible and safe option for diagnostic confirmation. In our case, intra-operative biopsy was performed without complications, nonetheless the massive adhesions of the ectopic splenic tissue with the surrounding organs and the asymptomatic status of the patient led to the decision of withholding surgical approach.

There is general consensus that treatment of splenosis should be reserved for symptomatic cases, while incidental lesions do not generally require therapeutic intervention (18). Surgical treatment, when feasible, is indicated in patients presenting with pain, bleeding, obstructive complications or recurrence of hematological conditions. In case of malignant disease suspect, it is more reasonable for the patient to undergo a laparotomic approach; if, on the other hand, the predominantly symptoms are chronic pelvic pain or dysmenorrhea without oncological risk factors, the laparoscopic approach is more advisable. Complete removal of splenic nodules is essential to avoid recurrence of the condition (19). To our knowledge, no published study in the literature directly compares the outcomes of patients with splenosis treated via laparotomy vs. laparoscopy.

Of note, although the degree of function of the splenosis ectopic splenic tissue remains often unknown, it has been documented that splenosis may be immunologically viable (20). Its functionality can be evidenced by peripheral smears lacking Howell-Jolly and Heinz bodies, which represent RBC remnants. The residual splenic tissue might be sufficient to restore its physiological function and potentially affect hematological diseases (18). Although rare, recurrence of hematological conditions in patients with splenosis after splenectomy have been reported in the literature (21). However, our patient was completely asymptomatic and did not show any hematological recurrences such as cytopenias or hemolysis. It could be hypothesized that, due to the blood supply being markedly different from the physiological vascularization of the spleen via the splenic artery, the newly formed splenic tissue may not fully perform its hemocatheretic function.

## Conclusion

Although it is an uncommon condition, splenosis should be considered as part of the differential diagnosis spectrum in patients presenting with an abdominal or pelvic mass and a previous history of splenic trauma or splenectomy. It represents a benign condition which can be potentially detected and diagnosed through non-invasive methods and patient's past medical history. Management in young female patients requires caution due to the highest risk of misdiagnosis and potential infertility complications. It should be noted that the growth of ectopic splenic tissue is typically slow and does not usually cause complications. Treatment of splenosis can be postponed until symptoms arise, opting for the laparoscopic approach in cases necessitating surgical intervention. Except for those rare symptomatic cases in which surgery is curative, a long-term clinical and radiological follow-up should be established after the diagnosis of splenosis. Gynecological follow-up for pelvic locations must be even more careful in young women of childbearing age.

## Data Availability

The raw data supporting the conclusions of this article will be made available by the authors, without undue reservation.
